# Supplementary material for: A gene-specific T2A-GAL4 library for Drosophila
Source: eLife. 2018 Mar 22;7:e35574. doi: 10.7554/eLife.35574 (PMC5898912; doi:10.7554/eLife.35574)
Supplement: Supplementary file 1. [file elife-35574-supp1.pptx]

## Slide 1
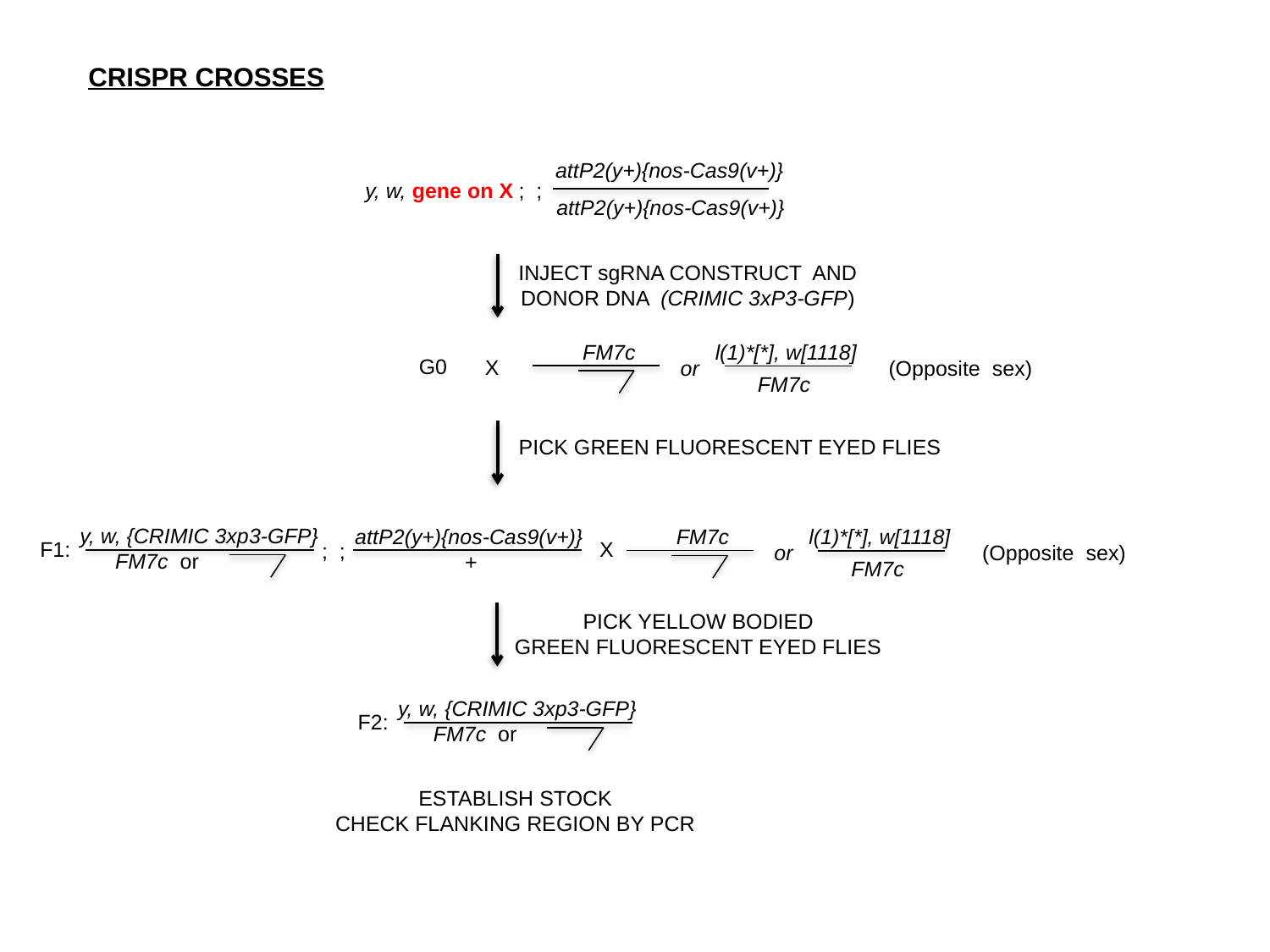

CRISPR CROSSES
attP2(y+){nos-Cas9(v+)}
y, w, gene on X
; ;
attP2(y+){nos-Cas9(v+)}
INJECT sgRNA CONSTRUCT AND
DONOR DNA (CRIMIC 3xP3-GFP)
FM7c
l(1)*[*], w[1118]
G0
X
(Opposite sex)
or
FM7c
PICK GREEN FLUORESCENT EYED FLIES
y, w, {CRIMIC 3xp3-GFP}
 FM7c or
FM7c
 attP2(y+){nos-Cas9(v+)}
l(1)*[*], w[1118]
F1:
X
; ;
(Opposite sex)
or
+
FM7c
PICK YELLOW BODIED
GREEN FLUORESCENT EYED FLIES
y, w, {CRIMIC 3xp3-GFP}
 FM7c or
F2:
ESTABLISH STOCK
CHECK FLANKING REGION BY PCR

## Slide 2
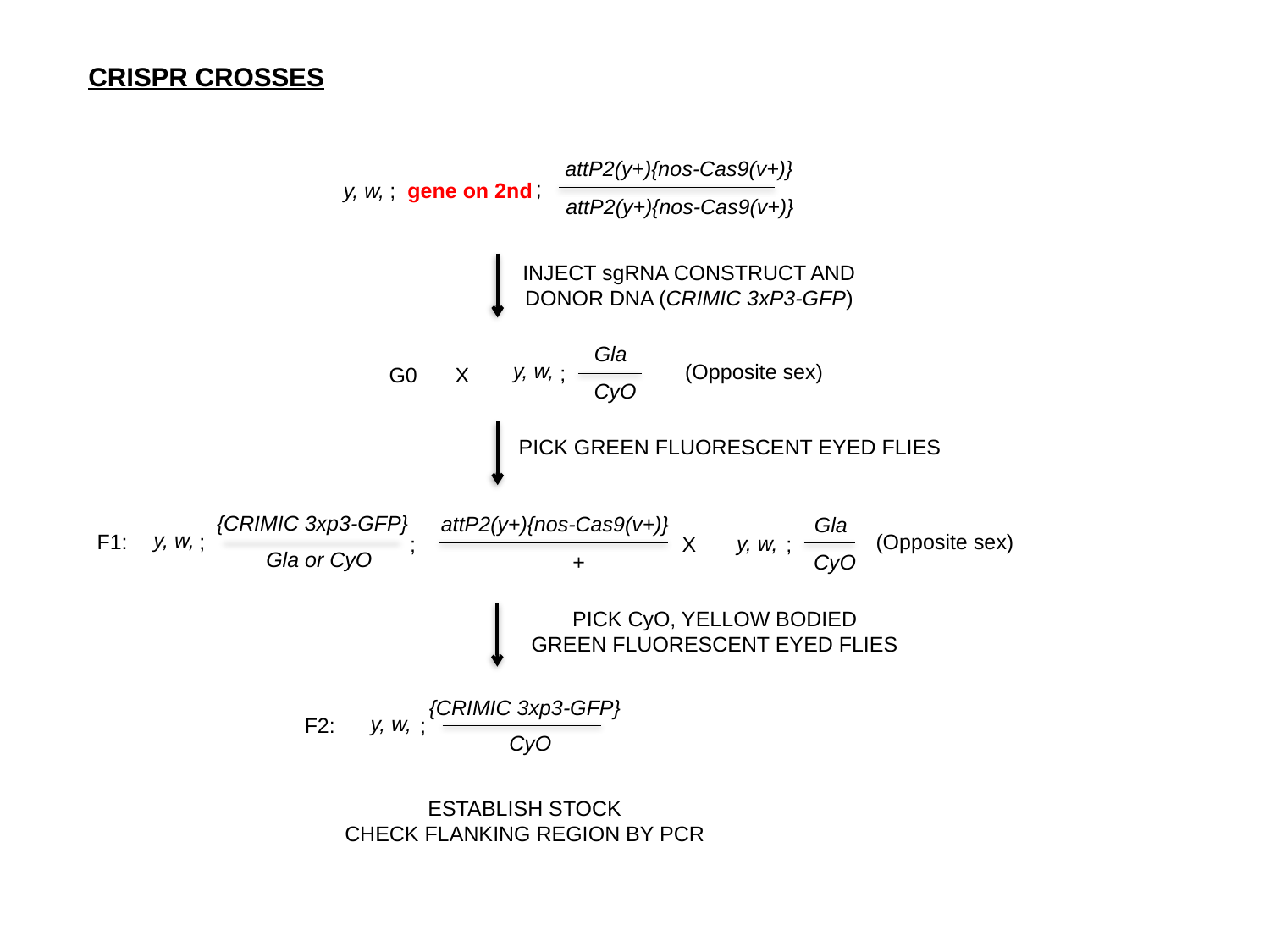

CRISPR CROSSES
attP2(y+){nos-Cas9(v+)}
;
; gene on 2nd
y, w,
attP2(y+){nos-Cas9(v+)}
INJECT sgRNA CONSTRUCT AND
DONOR DNA (CRIMIC 3xP3-GFP)
Gla
y, w,
(Opposite sex)
;
G0
X
 CyO
PICK GREEN FLUORESCENT EYED FLIES
 {CRIMIC 3xp3-GFP}
attP2(y+){nos-Cas9(v+)}
Gla
y, w,
(Opposite sex)
;
F1:
y, w,
X
;
;
Gla or CyO
CyO
+
PICK CyO, YELLOW BODIED
GREEN FLUORESCENT EYED FLIES
 {CRIMIC 3xp3-GFP}
y, w,
;
F2:
CyO
ESTABLISH STOCK
CHECK FLANKING REGION BY PCR

## Slide 3
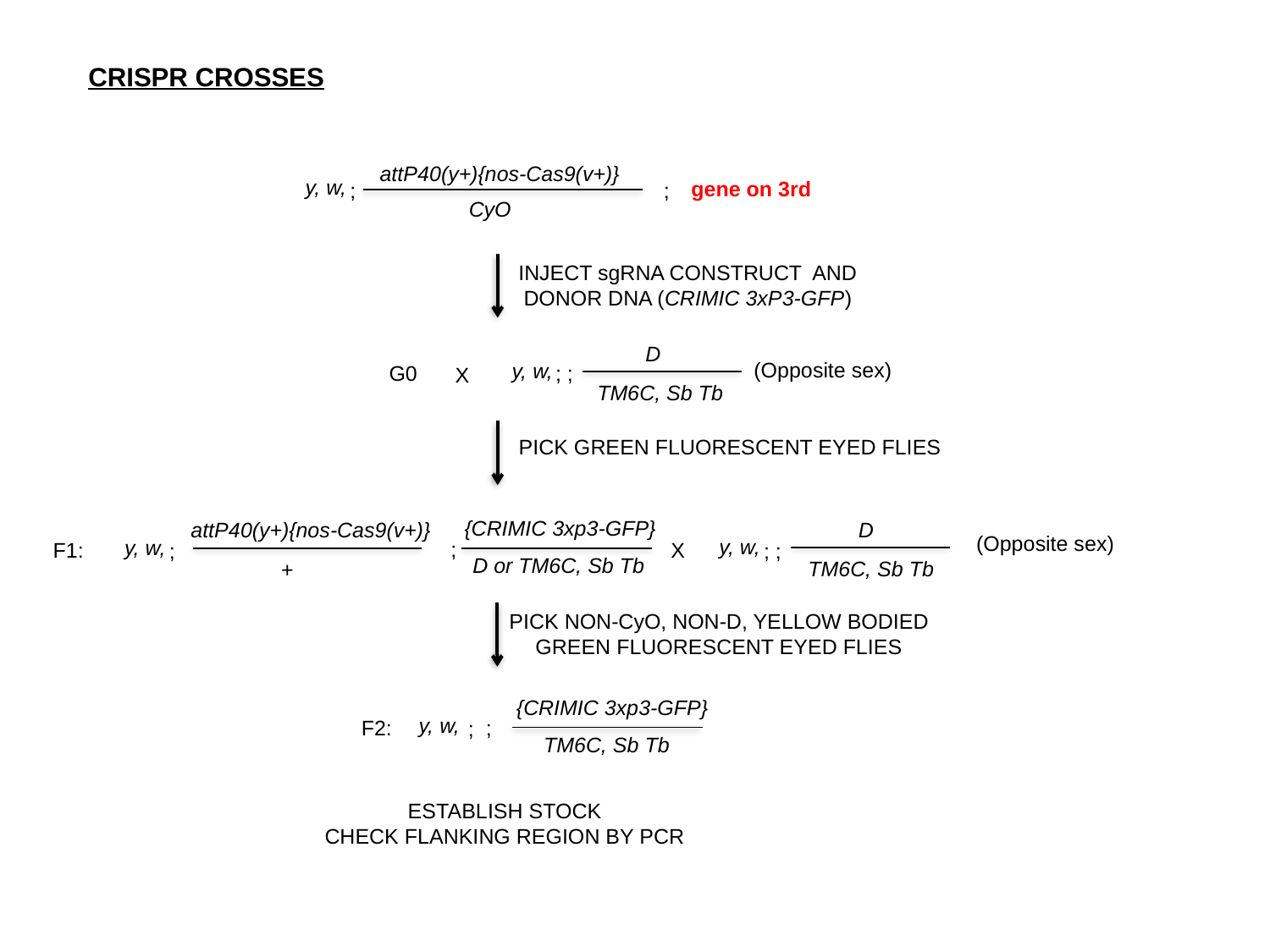

CRISPR CROSSES
attP40(y+){nos-Cas9(v+)}
y, w,
gene on 3rd
;
;
CyO
INJECT sgRNA CONSTRUCT AND
DONOR DNA (CRIMIC 3xP3-GFP)
D
(Opposite sex)
y, w,
; ;
G0
X
TM6C, Sb Tb
PICK GREEN FLUORESCENT EYED FLIES
 {CRIMIC 3xp3-GFP}
D
attP40(y+){nos-Cas9(v+)}
(Opposite sex)
y, w,
y, w,
;
X
F1:
; ;
;
D or TM6C, Sb Tb
TM6C, Sb Tb
+
PICK NON-CyO, NON-D, YELLOW BODIED
GREEN FLUORESCENT EYED FLIES
 {CRIMIC 3xp3-GFP}
y, w,
F2:
;
;
TM6C, Sb Tb
ESTABLISH STOCK
CHECK FLANKING REGION BY PCR

## Slide 4
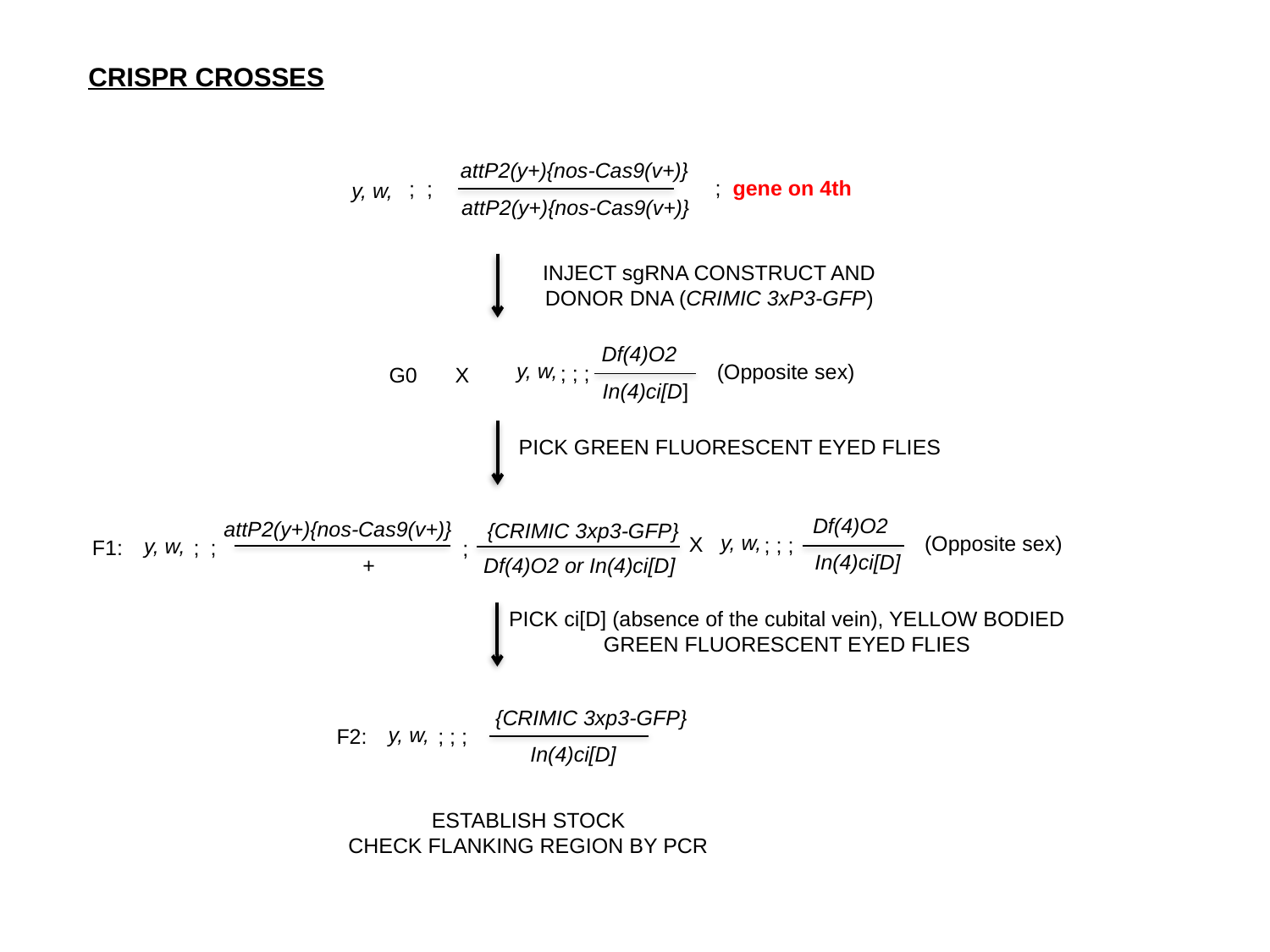

CRISPR CROSSES
attP2(y+){nos-Cas9(v+)}
; gene on 4th
; ;
y, w,
attP2(y+){nos-Cas9(v+)}
INJECT sgRNA CONSTRUCT AND
DONOR DNA (CRIMIC 3xP3-GFP)
Df(4)O2
y, w,
(Opposite sex)
; ; ;
G0
X
 In(4)ci[D]
PICK GREEN FLUORESCENT EYED FLIES
Df(4)O2
attP2(y+){nos-Cas9(v+)}
 {CRIMIC 3xp3-GFP}
y, w,
(Opposite sex)
X
; ; ;
y, w,
;
F1:
;
;
 In(4)ci[D]
 Df(4)O2 or In(4)ci[D]
+
PICK ci[D] (absence of the cubital vein), YELLOW BODIED
GREEN FLUORESCENT EYED FLIES
 {CRIMIC 3xp3-GFP}
y, w,
; ; ;
F2:
In(4)ci[D]
ESTABLISH STOCK
CHECK FLANKING REGION BY PCR
