# Supplementary material for: A gene-specific T2A-GAL4 library for Drosophila
Source: eLife. 2018 Mar 22;7:e35574. doi: 10.7554/eLife.35574 (PMC5898912; doi:10.7554/eLife.35574)
Supplement: Supplementary file 3. [file elife-35574-supp3.docx]

**DNA sequences of CRIMIC donor vectors.**

>pM14

CTAAATTGTAAGCGTTAATATTTTGTTAAAATTCGCGTTAAATTTTTGTTAAATCAGCTCATTTTTTAACCAATAGGCCGAAATCGGCAAAATCCCTTATAAATCAAAAGAATAGACCGAGATAGGGTTGAGTGTTGTTCCAGTTTGGAACAAGAGTCCACTATTAAAGAACGTGGACTCCAACGTCAAAGGGCGAAAAACCGTCTATCAGGGCGATGGCCCACTACGTGAACCATCACCCTAATCAAGTTTTTTGGGGTCGAGGTGCCGTAAAGCACTAAATCGGAACCCTAAAGGGAGCCCCCGATTTAGAGCTTGACGGGGAAAGCCGGCGAACGTGGCGAGAAAGGAAGGGAAGAAAGCGAAAGGAGCGGGCGCTAGGGCGCTGGCAAGTGTAGCGGTCACGCTGCGCGTAACCACCACACCCGCCGCGCTTAATGCGCCGCTACAGGGCGCGTCCCATTCGCCATTCAGGCTGCGCAACTGTTGGGAAGGGCGATCGGTGCGGGCCTCTTCGCTATTACGCCAGCTGGCGAAAGGGGGATGTGCTGCAAGGCGATTAAGTTGGGTAACGCCAGGGTTTTCCCAGTCACGACGTTGTAAAACGACGGCCAGTGAGCGCGCGTAATACGACTCACTATAGGGCGAATTGGAGCTCCACCGCGGTGGCGTCTCTAAACGGTCTCTAAACGAAGACTTAAACATGCATGTACTGACGGACACACCGAAGCCCCGGCGGCAACCCTCAGCGGATGCCCCGGGGCTTCACGTTTTCCCAGGTCAGAAGCGGTTTTCGGGAGTAGTGCCCCAACTGGGGTAACCTTTGAGTTCTCTCAGTTGGGGGCGTAGGGCCGCCGACATGACACAAGGGGTTGTGACCGGGGTGGACACGTACGCGGGTGCTTACGACCGTCAGTCGCGCGAGCGCGAGAAGTTCCTATTCTCTAGAAAGTATAGGAACTTCGAATTCAGTCGATCCAACATGGCGACTTGTCCCATCCCCGGCATGTTTAAATATACTAATTATTCTTGAACTAATTTTAATCAACCGATTTATCTCTCTTCCGCAGGTCTAACTAACTAACAAAGATCCAGACATGATAAGATACATTGATGAGTTTGGACAAACCACAACTAGAATGCAGTGAAAAAAATGCTTTATTTGTGAAATTTGTGATGCTATTGCTTTATTTGTAACCATTATAAGCTGCAATAAACAAGTTAACAACAACAATTGCATTCATTTTATGTTTCAGGTTCAGGGGGAGGTGTGGGAGGTTTTTTAAAGCAAGTAAAACCTCTACAAATGTGGTATGGCTGATTATGATCATAATTCGAGCTCGCCCGGGGATCTAATTCAATTAGAGACTAATTCAATTAGAGCTAATTCAATTAGGATCCAAGCTTATCGATTTCGAACCCTCGACCGCCGGAGTATAAATAGAGGCGCTTCGTCTACGGAGCGACAATTCAATTCAAACAAGCAAAGTGAACACGTCGCTAAGCGAAAGCTAAGCAAATAAACAAGCGCAGCTGAACAAGCTAAACAATCGGGGTACCGCTAGAGTCGACGGTACCGCGGGCCCGGGATCCACCGAGCGCCGGAGTATAAATAGAGGCGCTTCGTCTACGGAGCGACAATTCAATTCAAACAAGCAAAGTGAACACGTCGCTAAGCGAAAGCTAAGCAAATAAACAAGCGCAGCTGAACAAGCTAAACAATCTGCAGTAAAGTGCAAGTTAAAGTGAATCAATTAAAAGTAACCAGCAACCAAGTAAATCAACTGCAACTACTGAAATCTGCCAAGAAGTAATTATTGAATACAAGAAGAGAACTCTCGGCCGGCCACCATGGTGTCCAAGGGCGAGGAGCTGTTCACCGGCGTGGTGCCAATTCTGGTGGAGCTGGATGGCGACGTGAACGGCCACAAGTTCAGCGTGTCCGGCGAGGGCGAGGGCGACGCCACCTATGGAAAGCTGACCCTGAAGTTCATCTGCACCACCGGCAAGCTGCCCGTGCCATGGCCAACCCTCGTGACCACCCTGACCTATGGCGTGCAGTGCTTCAGCCGCTACCCCGATCACATGAAGCAGCACGATTTCTTCAAGAGCGCCATGCCCGAGGGCTACGTGCAGGAGCGCACCATCTTTTTCAAGGATGACGGCAACTACAAGACCCGCGCCGAAGTGAAGTTCGAGGGCGATACCCTCGTGAACCGCATCGAGCTGAAGGGCATCGATTTCAAGGAGGATGGAAACATCCTGGGCCACAAGCTGGAGTACAACTACAACAGCCACAACGTGTACATCATGGCCGACAAGCAGAAGAACGGCATCAAGGCCAACTTCAAGATCCGCCACAACATCGAGGATGGCGGCGTGCAGCTGGCCGATCACTACCAGCAGAACACCCCAATCGGCGACGGCCCAGTGCTGCTGCCCGATAACCATTACCTGAGCACCCAGAGCGCCCTGAGCAAGGATCCCAACGAGAAGCGCGACCACATGGTGCTGCTGGAGTTTGTGACCGCCGCCGGCATTACCCTGGGCATGGATGAGCTGTACAAGTAGGATCCAGACATGATAAGATACATTGATGAGTTTGGACAAACCACAACTAGAATGCAGTGAAAAAAATGCTTTATTTGTGAAATTTGTGATGCTATTGCTTTATTTGTAACCATTATAAGCTGCAATAAACAAGTTAACAACAACAATTGCATTCATTTTATGTTTCAGGTTCAGGGGGAGGTGTGGGAGGTTTTTTAAAGCAAGTAAAACCTCTACAAATGTGGTATGGCTGATTATGATCAGAAGTTCCTATTCTCTAGAAAGTATAGGAACTTCTCGCGCTCGCGCGACTGACGGTCGTAAGCACCCGCGTACGTGTCCACCCCGGTCACAACCCCTTGTGTCATGTCGGCGGCCCTACGCCCCCAACTGAGAGAACTCAAAGGTTACCCCAGTTGGGGCACTACTCCCGAAAACCGCTTCTGACCTGGGAAAACGTGAAGCCCCGGGGCATCCGCTGAGGGTTGCCGCCGGGGCTTCGGTGTGTCCGTCAGTACGCATGCCGCGTTGTCTTCCGCGTGAGACCCGCGTGAGACGGATATCAAGCTTATCGATACCGTCGACCTCGAGGGGGGGCCCGGTACCCAGCTTTTGTTCCCTTTAGTGAGGGTTAATTGCGCGCTTGGCGTAATCATGGTCATAGCTGTTTCCTGTGTGAAATTGTTATCCGCTCACAATTCCACACAACATACGAGCCGGAAGCATAAAGTGTAAAGCCTGGGGTGCCTAATGAGTGAGCTAACTCACATTAATTGCGTTGCGCTCACTGCCCGCTTTCCAGTCGGGAAACCTGTCGTGCCAGCTGCATTAATGAATCGGCCAACGCGCGGGGAGAGGCGGTTTGCGTATTGGGCGCTCTTCCGCTTCCTCGCTCACTGACTCGCTGCGCTCGGTCGTTCGGCTGCGGCGAGCGGTATCAGCTCACTCAAAGGCGGTAATACGGTTATCCACAGAATCAGGGGATAACGCAGGAAAGAACATGTGAGCAAAAGGCCAGCAAAAGGCCAGGAACCGTAAAAAGGCCGCGTTGCTGGCGTTTTTCCATAGGCTCCGCCCCCCTGACGAGCATCACAAAAATCGACGCTCAAGTCAGAGGTGGCGAAACCCGACAGGACTATAAAGATACCAGGCGTTTCCCCCTGGAAGCTCCCTCGTGCGCTCTCCTGTTCCGACCCTGCCGCTTACCGGATACCTGTCCGCCTTTCTCCCTTCGGGAAGCGTGGCGCTTTCTCATAGCTCACGCTGTAGGTATCTCAGTTCGGTGTAGGTCGTTCGCTCCAAGCTGGGCTGTGTGCACGAACCCCCCGTTCAGCCCGACCGCTGCGCCTTATCCGGTAACTATCGTCTTGAGTCCAACCCGGTAAGACACGACTTATCGCCACTGGCAGCAGCCACTGGTAACAGGATTAGCAGAGCGAGGTATGTAGGCGGTGCTACAGAGTTCTTGAAGTGGTGGCCTAACTACGGCTACACTAGAAGGACAGTATTTGGTATCTGCGCTCTGCTGAAGCCAGTTACCTTCGGAAAAAGAGTTGGTAGCTCTTGATCCGGCAAACAAACCACCGCTGGTAGCGGTGGTTTTTTTGTTTGCAAGCAGCAGATTACGCGCAGAAAAAAAGGATCTCAAGAAGATCCTTTGATCTTTTCTACGGGGTCTGACGCTCAGTGGAACGAAAACTCACGTTAAGGGATTTTGGTCATGAGATTATCAAAAAGGATCTTCACCTAGATCCTTTTAAATTAAAAATGAAGTTTTAAATCAATCTAAAGTATATATGAGTAAACTTGGTCTGACAGTTACCAATGCTTAATCAGTGAGGCACCTATCTCAGCGATCTGTCTATTTCGTTCATCCATAGTTGCCTGACTCCCCGTCGTGTAGATAACTACGATACGGGAGGGCTTACCATCTGGCCCCAGTGCTGCAATGATACCGCGtGACCCACGCTCACCGGCTCCAGATTTATCAGCAATAAACCAGCCAGCCGGAAGGGCCGAGCGCAGAAGTGGTCCTGCAACTTTATCCGCCTCCATCCAGTCTATTAATTGTTGCCGGGAAGCTAGAGTAAGTAGTTCGCCAGTTAATAGTTTGCGCAACGTTGTTGCCATTGCTACAGGCATCGTGGTGTCACGCTCGTCGTTTGGTATGGCTTCATTCAGCTCCGGTTCCCAACGATCAAGGCGAGTTACATGATCCCCCATGTTGTGCAAAAAAGCGGTTAGCTCCTTCGGTCCTCCGATCGTTGTCAGAAGTAAGTTGGCCGCAGTGTTATCACTCATGGTTATGGCAGCACTGCATAATTCTCTTACTGTCATGCCATCCGTAAGATGCTTTTCTGTGACTGGTGAGTACTCAACCAAGTCATTCTGAGAATAGTGTATGCGGCGACCGAGTTGCTCTTGCCCGGCGTCAATACGGGATAATACCGCGCCACATAGCAGAACTTTAAAAGTGCTCATCATTGGAAAACGTTCTTCGGGGCGAAAACTCTCAAGGATCTTACCGCTGTTGAGATCCAGTTCGATGTAACCCACTCGTGCACCCAACTGATCTTCAGCATCTTTTACTTTCACCAGCGTTTCTGGGTGAGCAAAAACAGGAAGGCAAAATGCCGCAAAAAAGGGAATAAGGGCGACACGGAAATGTTGAATACTCATACTCTTCCTTTTTCAATATTATTGAAGCATTTATCAGGGTTATTGTCTCATGAGCGGATACATATTTGAATGTATTTAGAAAAATAAACAAATAGGGGTTCCGCGCACATTTCCCCGAAAAGTGCCAC

>pM36

CTAAATTGTAAGCGTTAATATTTTGTTAAAATTCGCGTTAAATTTTTGTTAAATCAGCTCATTTTTTAACCAATAGGCCGAAATCGGCAAAATCCCTTATAAATCAAAAGAATAGACCGAGATAGGGTTGAGTGTTGTTCCAGTTTGGAACAAGAGTCCACTATTAAAGAACGTGGACTCCAACGTCAAAGGGCGAAAAACCGTCTATCAGGGCGATGGCCCACTACGTGAACCATCACCCTAATCAAGTTTTTTGGGGTCGAGGTGCCGTAAAGCACTAAATCGGAACCCTAAAGGGAGCCCCCGATTTAGAGCTTGACGGGGAAAGCCGGCGAACGTGGCGAGAAAGGAAGGGAAGAAAGCGAAAGGAGCGGGCGCTAGGGCGCTGGCAAGTGTAGCGGTCACGCTGCGCGTAACCACCACACCCGCCGCGCTTAATGCGCCGCTACAGGGCGCGTCCCATTCGCCATTCAGGCTGCGCAACTGTTGGGAAGGGCGATCGGTGCGGGCCTCTTCGCTATTACGCCAGCTGGCGAAAGGGGGATGTGCTGCAAGGCGATTAAGTTGGGTAACGCCAGGGTTTTCCCAGTCACGACGTTGTAAAACGACGGCCAGTGAGCGCGCGTAATACGACTCACTATAGGGCGAATTGGAGCTCCACCGCGGTGGCGTCTCTAACGGTCTCTAACGAAGACTTAAACATGCATCGATCGTCTGGTACTACATTCACGCGTACTGACGGACACACCGAAGCcccGGCGGCAACCCTCAGCGGATGCCCCGGGGCTTCACGTTTTCCCAGGTCAGAAGCGGTTTTCGGGAGTAGTGCCCCAACTGGGGTAACCTTTGAGTTCTCTCAGTTGGGGGCGTAGGGCCGCCGACATGACACAAGGGGTTGTGACCGGGGTGGACACGTACGCGGGTGCTTACGACCGTCAGTCGCGCGAGCGCGAGAATTCAGTCGATCCAACATGGCGACTTGTCCCATCCCCGGCATGTTTAAATATACTAATTATTCTTGAACTAATTTTAATCAACCGATTTATCTCTCTTCCGCAGGTCTAACTAACTAACAAAGATCCAGACATGATAAGATACATTGATGAGTTTGGACAAACCACAACTAGAATGCAGTGAAAAAAATGCTTTATTTGTGAAATTTGTGATGCTATTGCTTTATTTGTAACCATTATAAGCTGCAATAAACAAGTTAACAACAACAATTGCATTCATTTTATGTTTCAGGTTCAGGGGGAGGTGTGGGAGGTTTTTTAAAGCAAGTAAAACCTCTACAAATGTGGTATGGCTGATTATGATCATAATTCGAGCTCGCCCGGGGATCTAATTCAATTAGAGACTAATTCAATTAGAGCTAATTCAATTAGGATCCAAGCTTATCGATTTCGAACCCTCGACCGCCGGAGTATAAATAGAGGCGCTTCGTCTACGGAGCGACAATTCAATTCAAACAAGCAAAGTGAACACGTCGCTAAGCGAAAGCTAAGCAAATAAACAAGCGCAGCTGAACAAGCTAAACAATCGGGGTACCGCTAGAGTCGACGGTACCGCGGGCCCGGGATCCACCGAGCGCCGGAGTATAAATAGAGGCGCTTCGTCTACGGAGCGACAATTCAATTCAAACAAGCAAAGTGAACACGTCGCTAAGCGAAAGCTAAGCAAATAAACAAGCGCAGCTGAACAAGCTAAACAATCTGCAGTAAAGTGCAAGTTAAAGTGAATCAATTAAAAGTAACCAGCAACCAAGTAAATCAACTGCAACTACTGAAATCTGCCAAGAAGTAATTATTGAATACAAGAAGAGAACTCTCGGCCGGCCACCATGGTGTCCAAGGGCGAGGAGCTGTTCACCGGCGTGGTGCCAATTCTGGTGGAGCTGGATGGCGACGTGAACGGCCACAAGTTCAGCGTGTCCGGCGAGGGCGAGGGCGACGCCACCTATGGAAAGCTGACCCTGAAGTTCATCTGCACCACCGGCAAGCTGCCCGTGCCATGGCCAACCCTCGTGACCACCCTGACCTATGGCGTGCAGTGCTTCAGCCGCTACCCCGATCACATGAAGCAGCACGATTTCTTCAAGAGCGCCATGCCCGAGGGCTACGTGCAGGAGCGCACCATCTTTTTCAAGGATGACGGCAACTACAAGACCCGCGCCGAAGTGAAGTTCGAGGGCGATACCCTCGTGAACCGCATCGAGCTGAAGGGCATCGATTTCAAGGAGGATGGAAACATCCTGGGCCACAAGCTGGAGTACAACTACAACAGCCACAACGTGTACATCATGGCCGACAAGCAGAAGAACGGCATCAAGGCCAACTTCAAGATCCGCCACAACATCGAGGATGGCGGCGTGCAGCTGGCCGATCACTACCAGCAGAACACCCCAATCGGCGACGGCCCAGTGCTGCTGCCCGATAACCATTACCTGAGCACCCAGAGCGCCCTGAGCAAGGATCCCAACGAGAAGCGCGACCACATGGTGCTGCTGGAGTTTGTGACCGCCGCCGGCATTACCCTGGGCATGGATGAGCTGTACAAGTAGGATCCAGACATGATAAGATACATTGATGAGTTTGGACAAACCACAACTAGAATGCAGTGAAAAAAATGCTTTATTTGTGAAATTTGTGATGCTATTGCTTTATTTGTAACCATTATAAGCTGCAATAAACAAGTTAACAACAACAATTGCATTCATTTTATGTTTCAGGTTCAGGGGGAGGTGTGGGAGGTTTTTTAAAGCAAGTAAAACCTCTACAAATGTGGTATGGCTGATTATGATCATCGCGCTCGCGCGACTGACGGTCGTAAGCACCCGCGTACGTGTCCACCCCGGTCACAACCCCTTGTGTCATGTCGGCGGCCCTACGCCCCCAACTGAGAGAACTCAAAGGTTACCCCAGTTGGGGCACTACTCCCGAAAACCGCTTCTGACCTGGGAAAACGTGAAGCCCCGGGGCATCCGCTGAGGGTTGCCGCCGGGGCTTCGGTGTGTCCGTCAGTACGGTACTACCTCTCGTCCTTCACTGCGCATGCCGCGTTGTCTTCCGCGTGAGACCCGCGTGAGACGGATATCAAGCTTATCGATACCGTCGACCTCGAGGGGGGGCCCGGTACCCAGCTTTTGTTCCCTTTAGTGAGGGTTAATTGCGCGCTTGGCGTAATCATGGTCATAGCTGTTTCCTGTGTGAAATTGTTATCCGCTCACAATTCCACACAACATACGAGCCGGAAGCATAAAGTGTAAAGCCTGGGGTGCCTAATGAGTGAGCTAACTCACATTAATTGCGTTGCGCTCACTGCCCGCTTTCCAGTCGGGAAACCTGTCGTGCCAGCTGCATTAATGAATCGGCCAACGCGCGGGGAGAGGCGGTTTGCGTATTGGGCGCTCTTCCGCTTCCTCGCTCACTGACTCGCTGCGCTCGGTCGTTCGGCTGCGGCGAGCGGTATCAGCTCACTCAAAGGCGGTAATACGGTTATCCACAGAATCAGGGGATAACGCAGGAAAGAACATGTGAGCAAAAGGCCAGCAAAAGGCCAGGAACCGTAAAAAGGCCGCGTTGCTGGCGTTTTTCCATAGGCTCCGCCCCCCTGACGAGCATCACAAAAATCGACGCTCAAGTCAGAGGTGGCGAAACCCGACAGGACTATAAAGATACCAGGCGTTTCCCCCTGGAAGCTCCCTCGTGCGCTCTCCTGTTCCGACCCTGCCGCTTACCGGATACCTGTCCGCCTTTCTCCCTTCGGGAAGCGTGGCGCTTTCTCATAGCTCACGCTGTAGGTATCTCAGTTCGGTGTAGGTCGTTCGCTCCAAGCTGGGCTGTGTGCACGAACCCCCCGTTCAGCCCGACCGCTGCGCCTTATCCGGTAACTATCGTCTTGAGTCCAACCCGGTAAGACACGACTTATCGCCACTGGCAGCAGCCACTGGTAACAGGATTAGCAGAGCGAGGTATGTAGGCGGTGCTACAGAGTTCTTGAAGTGGTGGCCTAACTACGGCTACACTAGAAGGACAGTATTTGGTATCTGCGCTCTGCTGAAGCCAGTTACCTTCGGAAAAAGAGTTGGTAGCTCTTGATCCGGCAAACAAACCACCGCTGGTAGCGGTGGTTTTTTTGTTTGCAAGCAGCAGATTACGCGCAGAAAAAAAGGATCTCAAGAAGATCCTTTGATCTTTTCTACGGGGTCTGACGCTCAGTGGAACGAAAACTCACGTTAAGGGATTTTGGTCATGAGATTATCAAAAAGGATCTTCACCTAGATCCTTTTAAATTAAAAATGAAGTTTTAAATCAATCTAAAGTATATATGAGTAAACTTGGTCTGACAGTTACCAATGCTTAATCAGTGAGGCACCTATCTCAGCGATCTGTCTATTTCGTTCATCCATAGTTGCCTGACTCCCCGTCGTGTAGATAACTACGATACGGGAGGGCTTACCATCTGGCCCCAGTGCTGCAATGATACCGCGtGACCCACGCTCACCGGCTCCAGATTTATCAGCAATAAACCAGCCAGCCGGAAGGGCCGAGCGCAGAAGTGGTCCTGCAACTTTATCCGCCTCCATCCAGTCTATTAATTGTTGCCGGGAAGCTAGAGTAAGTAGTTCGCCAGTTAATAGTTTGCGCAACGTTGTTGCCATTGCTACAGGCATCGTGGTGTCACGCTCGTCGTTTGGTATGGCTTCATTCAGCTCCGGTTCCCAACGATCAAGGCGAGTTACATGATCCCCCATGTTGTGCAAAAAAGCGGTTAGCTCCTTCGGTCCTCCGATCGTTGTCAGAAGTAAGTTGGCCGCAGTGTTATCACTCATGGTTATGGCAGCACTGCATAATTCTCTTACTGTCATGCCATCCGTAAGATGCTTTTCTGTGACTGGTGAGTACTCAACCAAGTCATTCTGAGAATAGTGTATGCGGCGACCGAGTTGCTCTTGCCCGGCGTCAATACGGGATAATACCGCGCCACATAGCAGAACTTTAAAAGTGCTCATCATTGGAAAACGTTCTTCGGGGCGAAAACTCTCAAGGATCTTACCGCTGTTGAGATCCAGTTCGATGTAACCCACTCGTGCACCCAACTGATCTTCAGCATCTTTTACTTTCACCAGCGTTTCTGGGTGAGCAAAAACAGGAAGGCAAAATGCCGCAAAAAAGGGAATAAGGGCGACACGGAAATGTTGAATACTCATACTCTTCCTTTTTCAATATTATTGAAGCATTTATCAGGGTTATTGTCTCATGAGCGGATACATATTTGAATGTATTTAGAAAAATAAACAAATAGGGGTTCCGCGCACATTTCCCCGAAAAGTGCCAC

>pM37_p0

CTAAATTGTAAGCGTTAATATTTTGTTAAAATTCGCGTTAAATTTTTGTTAAATCAGCTCATTTTTTAACCAATAGGCCGAAATCGGCAAAATCCCTTATAAATCAAAAGAATAGACCGAGATAGGGTTGAGTGTTGTTCCAGTTTGGAACAAGAGTCCACTATTAAAGAACGTGGACTCCAACGTCAAAGGGCGAAAAACCGTCTATCAGGGCGATGGCCCACTACGTGAACCATCACCCTAATCAAGTTTTTTGGGGTCGAGGTGCCGTAAAGCACTAAATCGGAACCCTAAAGGGAGCCCCCGATTTAGAGCTTGACGGGGAAAGCCGGCGAACGTGGCGAGAAAGGAAGGGAAGAAAGCGAAAGGAGCGGGCGCTAGGGCGCTGGCAAGTGTAGCGGTCACGCTGCGCGTAACCACCACACCCGCCGCGCTTAATGCGCCGCTACAGGGCGCGTCCCATTCGCCATTCAGGCTGCGCAACTGTTGGGAAGGGCGATCGGTGCGGGCCTCTTCGCTATTACGCCAGCTGGCGAAAGGGGGATGTGCTGCAAGGCGATTAAGTTGGGTAACGCCAGGGTTTTCCCAGTCACGACGTTGTAAAACGACGGCCAGTGAGCGCGCGTAATACGACTCACTATAGGGCGAATTGGAGCTCCACCGCGGTGGCGTCTCTAAACGGTCTCTAAACGAAGACTTAAACATGCATGTACTGACGGACACACCGAAGCCCCGGCGGCAACCCTCAGCGGATGCCCCGGGGCTTCACGTTTTCCCAGGTCAGAAGCGGTTTTCGGGAGTAGTGCCCCAACTGGGGTAACCTTTGAGTTCTCTCAGTTGGGGGCGTAGGGCCGCCGACATGACACAAGGGGTTGTGACCGGGGTGGACACGTACGCGGGTGCTTACGACCGTCAGTCGCGCGAGCGCGAGAAGTTCCTATTCTCTAGAAAGTATAGGAACTTCGAATTGAGTCGATCCAACATGGCGACTTGTCCCATCCCCGGCATGTTTAAATATACTAATTATTCTTGAACTAATTTTAATCAACCGATTTATCTCTCTTCCGCAGGTGGGAGGTTCCGGTGGAAGCGGAGGTAGCGGCACCGGTGCATGCGAGGGCCGCGGCAGCCTGCTGACCTGCGGCGATGTGGAGGAGAACCCCGGGCCCATGAAGCTGCTGAGCAGCATCGAGCAGGCCTGCGATATCTGCCGCCTGAAGAAGCTGAAGTGCAGCAAGGAGAAGCCCAAGTGCGCCAAGTGCCTGAAGAACAACTGGGAGTGCCGCTACAGCCCCAAGACCAAGCGCAGCCCCCTGACCCGCGCCCACCTGACCGAGGTGGAGAGCCGCCTGGAGCGCCTGGAGCAGCTGTTCCTGCTGATCTTCCCCCGCGAGGATCTGGATATGATCCTGAAGATGGATAGCCTGCAGGATATCAAGGCCCTGCTGACCGGCCTGTTCGTGCAGGATAACGTGAACAAGGATGCCGTGACCGATCGCCTGGCCAGCGTGGAAACCGATATGCCCCTGACCCTGCGCCAGCACCGCATCAGCGCCACCAGCAGCAGCGAGGAGAGCAGCAACAAGGGCCAGCGCCAGCTGACCGTGAGCATCGATAGCGCCGCCCACCACGATAACAGCACCATCCCCCTGGATTTCATGCCCCGCGATGCCCTGCACGGCTTCGATTGGAGCGAGGAGGATGATATGAGCGATGGCCTGCCCTTCCTGAAAACCGATCCCAACAACAACGGCTTCTTCGGCGATGGCAGCCTGCTGTGCATCCTGCGCAGCATCGGCTTCAAGCCCGAGAACTACACCAACAGCAACGTGAACCGCCTGCCCACCATGATCACCGATCGCTACACCCTGGCCAGCCGCAGCACCACCAGCCGCCTGCTGCAGAGCTACCTGAACAACTTCCACCCCTACTGCCCCATCGTGCACAGCCCCACCCTGATGATGCTGTACAACAACCAGATCGAGATCGCCAGCAAGGATCAGTGGCAGATCCTGTTCAACTGCATCCTGGCCATCGGCGCCTGGTGCATCGAGGGCGAGAGCACCGATATCGATGTGTTCTACTACCAGAACGCCAAGAGCCACCTGACCAGCAAGGTGTTCGAGAGCGGCAGCATCATCCTGGTGACCGCCCTGCACCTGCTGAGCCGCTACACCCAGTGGCGCCAAAAGACCAACACCAGCTACAACTTCCACAGCTTCAGCATCCGCATGGCCATCAGCCTGGGCCTGAACCGCGATCTGCCCAGCAGCTTCAGCGATAGCAGCATCCTGGAGCAGCGCCGCCGCATCTGGTGGAGCGTGTACAGCTGGGAGATCCAGCTGAGCCTGCTGTACGGCCGCAGCATCCAGCTGAGCCAGAACACCATCAGCTTCCCCAGCAGCGTGGATGATGTGCAGCGCACCACCACCGGCCCCACCATCTACCACGGCATCATCGAGACTGCCCGCCTGCTGCAGGTGTTCACCAAGATCTACGAGCTGGATAAGACCGTGACCGCCGAGAAGAGCCCCATCTGCGCCAAGAAGTGCCTGATGATCTGCAACGAGATCGAGGAGGTGAGCCGCCAGGCCCCCAAGTTCCTGCAGATGGATATCAGCACCACCGCCCTGACCAACCTGCTGAAGGAGCACCCCTGGCTGAGCTTCACCCGCTTCGAGCTGAAGTGGAAGCAGCTGAGCCTGATCATCTACGTGCTGCGCGATTTCTTCACCAACTTCACCCAGAAGAAGAGCCAGCTGGAGCAGGATCAGAACGATCACCAGAGCTACGAGGTGAAGCGCTGCAGCATCATGCTGAGCGATGCCGCCCAGCGCACCGTGATGAGCGTGAGCAGCTACATGGATAACCACAACGTGACCCCCTACTTCGCCTGGAACTGCAGCTACTACCTGTTCAACGCCGTGCTGGTGCCCATCAAGACCCTGCTGAGCAACAGCAAGAGCAACGCCGAGAACAACGAAACGGCCCAGCTGCTGCAGCAGATCAACACCGTGCTGATGCTGCTGAAGAAGCTGGCCACCTTCAAGATCCAGACCTGCGAGAAGTACATCCAGGTGCTGGAGGAGGTGTGCGCCCCCTTCCTGCTGAGCCAGTGCGCCATCCCCCTGCCCCACATCAGCTACAACAACAGCAACGGCAGCGCCATCAAGAACATCGTGGGCAGCGCCACCATCGCCCAGTACCCCACCCTGCCCGAGGAGAACGTGAACAACATCAGCGTGAAGTACGTGAGCCCCGGCAGCGTGGGACCCAGCCCCGTGCCCCTGAAGAGCGGCGCCAGCTTCAGCGATCTGGTGAAGCTGCTGAGCAACCGCCCCCCCAGCCGCAACAGCCCCGTGACCATCCCCCGCAGCACCCCCAGCCACCGCAGCGTGACCCCCTTCCTGGGCCAGCAGCAGCAGCTGCAGAGCCTGGTGCCCCTGACCCCCAGCGCCCTGTTCGGCGGCGCCAACTTCAACCAGAGCGGCAACATCGCCGATAGCAGCCTGAGCTTCACCTTCACCAACAGCAGCAACGGCCCCAACCTGATCACCACCCAGACCAACAGCCAGGCCCTGAGCCAGCCCATCGCCAGCAGCAACGTGCACGATAACTTCATGAACAACGAGATCACCGCCAGCAAGATCGATGATGGCAACAACAGCAAGCCCCTGAGCCCCGGCTGGACCGATCAGACCGCCTACAACGCCTTCGGCATCACCACCGGCATGTTCAACACCACCACCATGGATGATGTGTACAACTACCTGTTCGATGATGAGGATACCCCCCCCAACCCCAAGAAGGAGTAATAAGGCGCGCCCAAAGATCCAGACATGATAAGATACATTGATGAGTTTGGACAAACCACAACTAGAATGCAGTGAAAAAAATGCTTTATTTGTGAAATTTGTGATGCTATTGCTTTATTTGTAACCATTATAAGCTGCAATAAACAAGTTAACAACAACAATTGCATTCATTTTATGTTTCAGGTTCAGGGGGAGGTGTGGGAGGTTTTTTAAAGCAAGTAAAACCTCTACAAATGTGGTATGGCTGATTATGATCATAATTCGAGCTCGCCCGGGGATCTAATTCAATTAGAGACTAATTCAATTAGAGCTAATTCAATTAGGATCCAAGCTTATCGATTTCGAACCCTCGACCGCCGGAGTATAAATAGAGGCGCTTCGTCTACGGAGCGACAATTCAATTCAAACAAGCAAAGTGAACACGTCGCTAAGCGAAAGCTAAGCAAATAAACAAGCGCAGCTGAACAAGCTAAACAATCGGACTAGAGCCGGTCGCCGGCCGGCCACCATGGTGTCCAAGGGCGAGGAGCTGTTCACCGGCGTGGTGCCAATTCTGGTGGAGCTGGATGGCGACGTGAACGGCCACAAGTTCAGCGTGTCCGGCGAGGGCGAGGGCGACGCCACCTATGGAAAGCTGACCCTGAAGTTCATCTGCACCACCGGCAAGCTGCCCGTGCCATGGCCAACCCTCGTGACCACCCTGACCTATGGCGTGCAGTGCTTCAGCCGCTACCCCGATCACATGAAGCAGCACGATTTCTTCAAGAGCGCCATGCCCGAGGGCTACGTGCAGGAGCGCACCATCTTTTTCAAGGATGACGGCAACTACAAGACCCGCGCCGAAGTGAAGTTCGAGGGCGATACCCTCGTGAACCGCATCGAGCTGAAGGGCATCGATTTCAAGGAGGATGGAAACATCCTGGGCCACAAGCTGGAGTACAACTACAACAGCCACAACGTGTACATCATGGCCGACAAGCAGAAGAACGGCATCAAGGCCAACTTCAAGATCCGCCACAACATCGAGGATGGCGGCGTGCAGCTGGCCGATCACTACCAGCAGAACACCCCAATCGGCGACGGCCCAGTGCTGCTGCCCGATAACCATTACCTGAGCACCCAGAGCGCCCTGAGCAAGGATCCCAACGAGAAGCGCGACCACATGGTGCTGCTGGAGTTTGTGACCGCCGCCGGCATTACCCTGGGCATGGATGAGCTGTACAAGTAGGATCCAGACATGATAAGATACATTGATGAGTTTGGACAAACCACAACTAGAATGCAGTGAAAAAAATGCTTTATTTGTGAAATTTGTGATGCTATTGCTTTATTTGTAACCATTATAAGCTGCAATAAACAAGTTAACAACAACAATTGCATTCATTTTATGTTTCAGGTTCAGGGGGAGGTGTGGGAGGTTTTTTAAAGCAAGTAAAACCTCTACAAATGTGGTATGGCTGATTATGATCAGAAGTTCCTATTCTCTAGAAAGTATAGGAACTTCTCGCGCTCGCGCGACTGACGGTCGTAAGCACCCGCGTACGTGTCCACCCCGGTCACAACCCCTTGTGTCATGTCGGCGGCCCTACGCCCCCAACTGAGAGAACTCAAAGGTTACCCCAGTTGGGGCACTACTCCCGAAAACCGCTTCTGACCTGGGAAAACGTGAAGCCCCGGGGCATCCGCTGAGGGTTGCCGCCGGGGCTTCGGTGTGTCCGTCAGTACGCATGCCGCGTTGTCTTCCGCGTGAGACCCGCGTGAGACGGATATCAAGCTTATCGATACCGTCGACCTCGAGGGGGGGCCCGGTACCCAGCTTTTGTTCCCTTTAGTGAGGGTTAATTGCGCGCTTGGCGTAATCATGGTCATAGCTGTTTCCTGTGTGAAATTGTTATCCGCTCACAATTCCACACAACATACGAGCCGGAAGCATAAAGTGTAAAGCCTGGGGTGCCTAATGAGTGAGCTAACTCACATTAATTGCGTTGCGCTCACTGCCCGCTTTCCAGTCGGGAAACCTGTCGTGCCAGCTGCATTAATGAATCGGCCAACGCGCGGGGAGAGGCGGTTTGCGTATTGGGCGCTCTTCCGCTTCCTCGCTCACTGACTCGCTGCGCTCGGTCGTTCGGCTGCGGCGAGCGGTATCAGCTCACTCAAAGGCGGTAATACGGTTATCCACAGAATCAGGGGATAACGCAGGAAAGAACATGTGAGCAAAAGGCCAGCAAAAGGCCAGGAACCGTAAAAAGGCCGCGTTGCTGGCGTTTTTCCATAGGCTCCGCCCCCCTGACGAGCATCACAAAAATCGACGCTCAAGTCAGAGGTGGCGAAACCCGACAGGACTATAAAGATACCAGGCGTTTCCCCCTGGAAGCTCCCTCGTGCGCTCTCCTGTTCCGACCCTGCCGCTTACCGGATACCTGTCCGCCTTTCTCCCTTCGGGAAGCGTGGCGCTTTCTCATAGCTCACGCTGTAGGTATCTCAGTTCGGTGTAGGTCGTTCGCTCCAAGCTGGGCTGTGTGCACGAACCCCCCGTTCAGCCCGACCGCTGCGCCTTATCCGGTAACTATCGTCTTGAGTCCAACCCGGTAAGACACGACTTATCGCCACTGGCAGCAGCCACTGGTAACAGGATTAGCAGAGCGAGGTATGTAGGCGGTGCTACAGAGTTCTTGAAGTGGTGGCCTAACTACGGCTACACTAGAAGGACAGTATTTGGTATCTGCGCTCTGCTGAAGCCAGTTACCTTCGGAAAAAGAGTTGGTAGCTCTTGATCCGGCAAACAAACCACCGCTGGTAGCGGTGGTTTTTTTGTTTGCAAGCAGCAGATTACGCGCAGAAAAAAAGGATCTCAAGAAGATCCTTTGATCTTTTCTACGGGGTCTGACGCTCAGTGGAACGAAAACTCACGTTAAGGGATTTTGGTCATGAGATTATCAAAAAGGATCTTCACCTAGATCCTTTTAAATTAAAAATGAAGTTTTAAATCAATCTAAAGTATATATGAGTAAACTTGGTCTGACAGTTACCAATGCTTAATCAGTGAGGCACCTATCTCAGCGATCTGTCTATTTCGTTCATCCATAGTTGCCTGACTCCCCGTCGTGTAGATAACTACGATACGGGAGGGCTTACCATCTGGCCCCAGTGCTGCAATGATACCGCGTGACCCACGCTCACCGGCTCCAGATTTATCAGCAATAAACCAGCCAGCCGGAAGGGCCGAGCGCAGAAGTGGTCCTGCAACTTTATCCGCCTCCATCCAGTCTATTAATTGTTGCCGGGAAGCTAGAGTAAGTAGTTCGCCAGTTAATAGTTTGCGCAACGTTGTTGCCATTGCTACAGGCATCGTGGTGTCACGCTCGTCGTTTGGTATGGCTTCATTCAGCTCCGGTTCCCAACGATCAAGGCGAGTTACATGATCCCCCATGTTGTGCAAAAAAGCGGTTAGCTCCTTCGGTCCTCCGATCGTTGTCAGAAGTAAGTTGGCCGCAGTGTTATCACTCATGGTTATGGCAGCACTGCATAATTCTCTTACTGTCATGCCATCCGTAAGATGCTTTTCTGTGACTGGTGAGTACTCAACCAAGTCATTCTGAGAATAGTGTATGCGGCGACCGAGTTGCTCTTGCCCGGCGTCAATACGGGATAATACCGCGCCACATAGCAGAACTTTAAAAGTGCTCATCATTGGAAAACGTTCTTCGGGGCGAAAACTCTCAAGGATCTTACCGCTGTTGAGATCCAGTTCGATGTAACCCACTCGTGCACCCAACTGATCTTCAGCATCTTTTACTTTCACCAGCGTTTCTGGGTGAGCAAAAACAGGAAGGCAAAATGCCGCAAAAAAGGGAATAAGGGCGACACGGAAATGTTGAATACTCATACTCTTCCTTTTTCAATATTATTGAAGCATTTATCAGGGTTATTGTCTCATGAGCGGATACATATTTGAATGTATTTAGAAAAATAAACAAATAGGGGTTCCGCGCACATTTCCCCGAAAAGTGCCAC

XXXX:attP site

XXXX:FRT site

XXXX:splice acceptor

XXXX:3xstop

XXXX:linker variable region*

XXXX:T2A-Gal4

XXXX:SV40 polyA

XXXX:3xP3-GFP

* For pM37_p1 linker sequence is GTGGCGGAGGTTCCGGTGGAAGCGGAGGTAGCGGCACTAGTACGCGT

* For pM37_p2 linker sequence is GTCGGGAGGTTCCGGTGGAAGCGGAGGTAGCGGCGAATTCCGTACG
